# CSF-net: a color space fusion network with self-attention-driven feature learning for feline ocular diseases classification

**DOI:** 10.3389/fvets.2026.1826139

**Published:** 2026-04-22

**Authors:** Dhivyaa S. P., Myungeun Lee, Hyung-Jeong Yang

**Affiliations:** 1 Department of Artificial Intelligence Convergence, Chonnam National University, Gwangju, Republic of Korea; 2 Hyper-Wide Federated Medical AI Research Center, Chonnam National University, Gwangju, Republic of Korea

**Keywords:** AI in veterinary medicine, deep learning, feline ocular disease, mobile AI diagnostics, multi-color space, veterinary ophthalmology

## Abstract

**Introduction:**

Feline ocular diseases can cause irreversible vision loss if they are not detected early. However, early diagnosis is often difficult. This is due to limited access to veterinary ophthalmology services and the challenge of distinguishing between visually similar eye conditions. Illumination changes, glare, and strong visual similarity among diseases substantially limit the performance of conventional RGB-based classification models. This paper proposes the Color Space Fusion Network (CSF-Net), an attention-guided color interaction framework for robust feline ocular disease classification in real-world environments.

**Methods:**

CSF-Net decomposes images into complementary color spaces (RGB, HSV, and YCbCr), learns independent feature representations, and applies a self-attention mechanism to emphasize disease-relevant visual cues while reducing sensitivity to environmental variations. The proposed method is evaluated on a large-scale dataset of 16,480 feline ocular images and compared with representative deep learning models, including ResNet50, EfficientNet, and ViT/16-b.

**Results:**

CSF-Net achieves superior performance, reaching an accuracy of 83.05% and achieved a mean fold-wise macro-AUC of 0.9690 across five cross-validation folds. Ablation and class-wise analyses confirm the effectiveness of self-attention in leveraging multi-color representations, while also revealing limitations for eyelid-centered conditions such as blepharitis. A mobile-based preliminary screening application, PurrfectEyes, is further presented to demonstrate practical applicability.

**Discussion:**

Overall, this work introduces an attention-guided multi-color framework that improves robustness and interpretability for AI-assisted feline ocular disease screening in real-world settings.

## 1 Introduction

Feline ocular diseases represent a significant challenge in veterinary medicine due to their adverse effects on vision, quality of life, and overall feline health (1). Many of these conditions progress rapidly and may lead to irreversible vision loss if not identified early (2, 3). However, timely diagnosis remains difficult in real-world settings because access to specialized veterinary ophthalmology services is limited, and pet owners often lack the expertise to recognize early ocular abnormalities (4, 5).

In addition to these practical constraints, diagnosis is further complicated by the substantial visual similarity among common feline ocular conditions (6). Disorders such as blepharitis, conjunctivitis, corneal sequestrum, non-ulcerative keratitis, and corneal ulcer often present overlapping clinical signs, including redness, tearing, discharge, corneal opacity, and ocular discomfort (7). While blepharitis primarily involves eyelid inflammation (8), conjunctivitis is associated with excessive tearing and discharge (9). Corneal sequestrum manifests as dark necrotic plaques on the cornea (10), non-ulcerative keratitis leads to corneal cloudiness without epithelial damage (11), and corneal ulcers involve corneal surface disruption accompanied by pain and photophobia (12). Because these symptoms are visually similar and often subtle at onset, pet owners frequently struggle to distinguish between conditions. This can lead to misinterpretation, delayed veterinary visits, or inappropriate home management.

Recent advances in artificial intelligence have demonstrated strong potential for medical image-based diagnosis, particularly in human ophthalmology, where deep learning models have achieved high accuracy in detecting retinal diseases (13, 14). Convolutional neural networks have been shown to effectively extract discriminative features from ophthalmic images (15), motivating their application to veterinary settings. However, models developed under controlled clinical conditions often fail to generalize to real-world scenarios. Images captured by pet owners exhibit significant variability due to illumination changes, shadows, glare, and differences in camera quality. These factors can degrade model performance. Furthermore, conventional approaches relying solely on RGB representations may not sufficiently capture the texture and contrast variations required for reliable disease differentiation under such conditions (5).

This paper proposes the Color Space Fusion Network (CSF-Net), a classification framework designed to address visual variability and inter-class ambiguity in real-world feline ocular images. CSF-Net decomposes input images into complementary color spaces (RGB, HSV, and YCbCr) and learns independent feature representations for each domain. A self-attention mechanism is then used to model cross-color feature interactions and selectively emphasize disease-relevant visual cues. This reduces sensitivity to illumination-induced artifacts and improves classification reliability. Unlike simple color-space expansion, the proposed approach introduces an attention-guided feature interaction framework tailored to unconstrained veterinary imaging conditions. The main contributions of this paper are summarized as follows:

- Problem formulation and model proposal: this work formulates feline ocular disease classification under real-world conditions as a problem influenced by illumination variability and inter-disease visual ambiguity. It proposes CSF-Net, an attention-guided multi-color space fusion framework designed to address these challenges.
- Attention-based multi-color feature interaction: CSF-Net decomposes RGB, HSV, and YCbCr representations into independent feature subspaces and employs self-attention to model cross-color interactions. This enables selective emphasis on disease-relevant visual cues beyond naive color-space concatenation.
- Experimental validation and analysis: extensive experiments on a large-scale real-world feline ocular disease dataset demonstrate that CSF-Net consistently outperforms representative deep learning models. Ablation studies further show that performance gains primarily arise from attention-guided feature interaction rather than color-space expansion alone.
- Interpretability and practical applicability: class-wise analysis and Grad-CAM visualizations provide clinically meaningful interpretation of model behavior. Integration into a mobile-based preliminary screening application further demonstrates practical feasibility as an assistive tool for early awareness and veterinary consultation.

## 2 Materials and methods

### 2.1 Dataset and preprocessing

**Figure 1:**
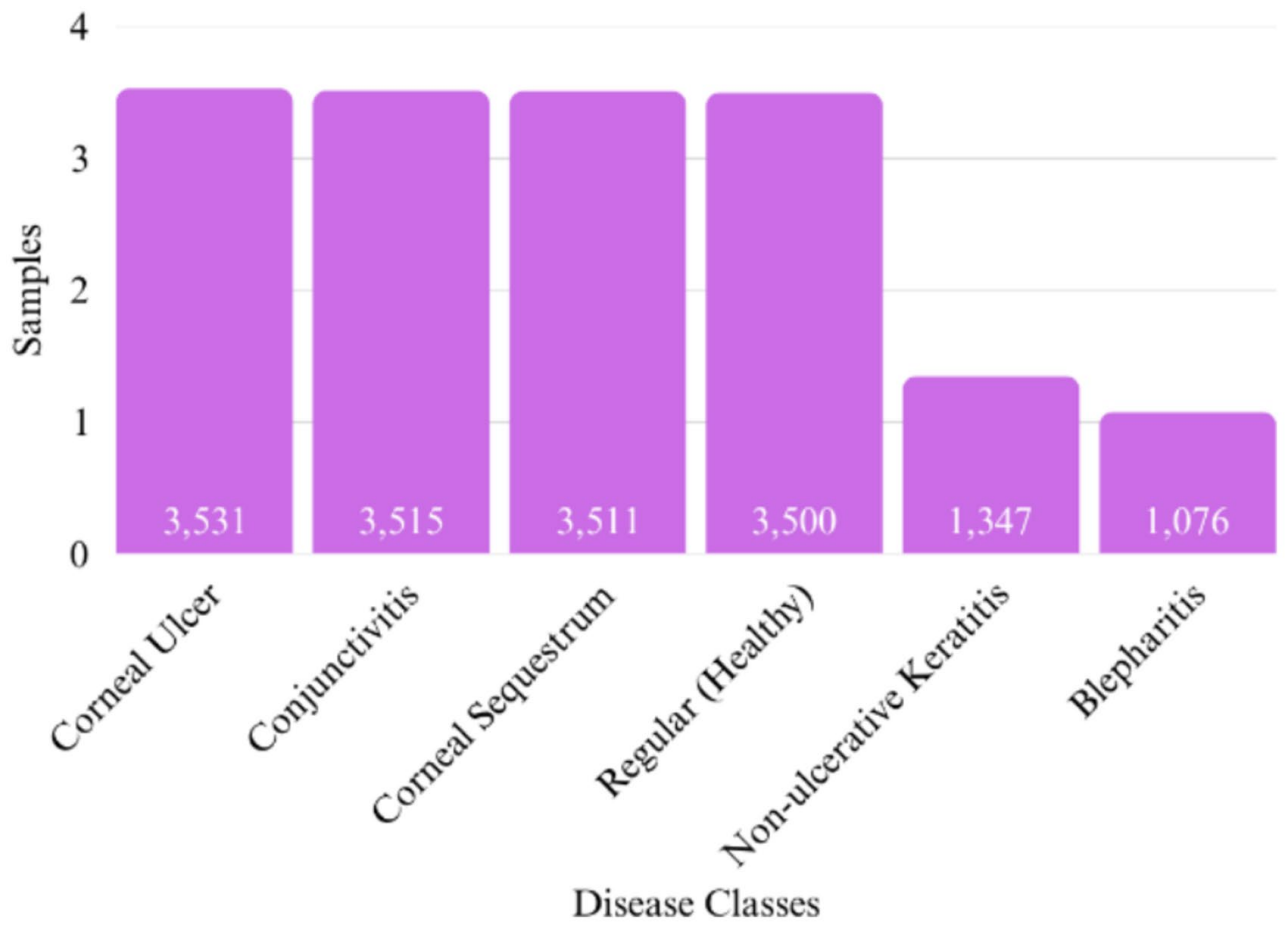
Dataset characteristics: disease class distribution & number of samples per disease category.

The Pet Eye Disease dataset from AI Hub (16), used in this study consists of 16,480 images categorized into six classes: Blepharitis, Conjunctivitis, Corneal Sequestrum, Non-ulcerative Keratitis, Corneal Ulcer, and Healthy Eyes. This dataset has been validated by three veterinary clinicians to ensure accurate labelling. Figure 1 presents the characteristics of the dataset, detailing the distribution of samples across different disease categories.

The dataset was collected using two types of imaging devices: ophthalmic instruments (15,392 images) and general cameras (1,088 images), across multiple clinical and non-clinical environments. For ophthalmic images, exclusion criteria included out-of-focus images, slit-lamp images, and full-face images, while for general camera images, constraints such as disabling auto-correction, avoiding zoom, and retaking out-of-focus images were enforced. These procedures help ensure a baseline level of quality while preserving real-world variability in imaging conditions. As the study is based on secondary analysis of anonymized image data and does not involve direct interaction with animals, it complies with applicable ethical standards for animal research. Detailed demographic distributions (age and breed) are provided in Supplementary Figure S1.

Disease categories were mapped to integer class labels, and samples with missing or unmapped labels were excluded. Stratified k-fold cross-validation was employed to preserve class distributions across training and validation splits. As subject-level identifiers are not available in the dataset, the splitting was performed at the image level. While duplicate images were removed during dataset construction, the possibility of multiple images from the same subject appearing across different folds cannot be fully excluded. Images were loaded using PIL, converted to RGB, and resized to 224 × 224 pixels. Training data augmentation included random horizontal flipping (*p* = 0.5), random rotation (±15°), and color jittering (brightness, contrast, and saturation = 0.2), while validation images were resized only. Each image was decomposed into RGB, HSV, and YCbCr color spaces. Channel-wise mean and standard deviation values were computed from the training set and used to normalize both training and validation images. All images were then converted to tensor representations for model input.

### 2.2 Proposed AI model

**Figure 2:**
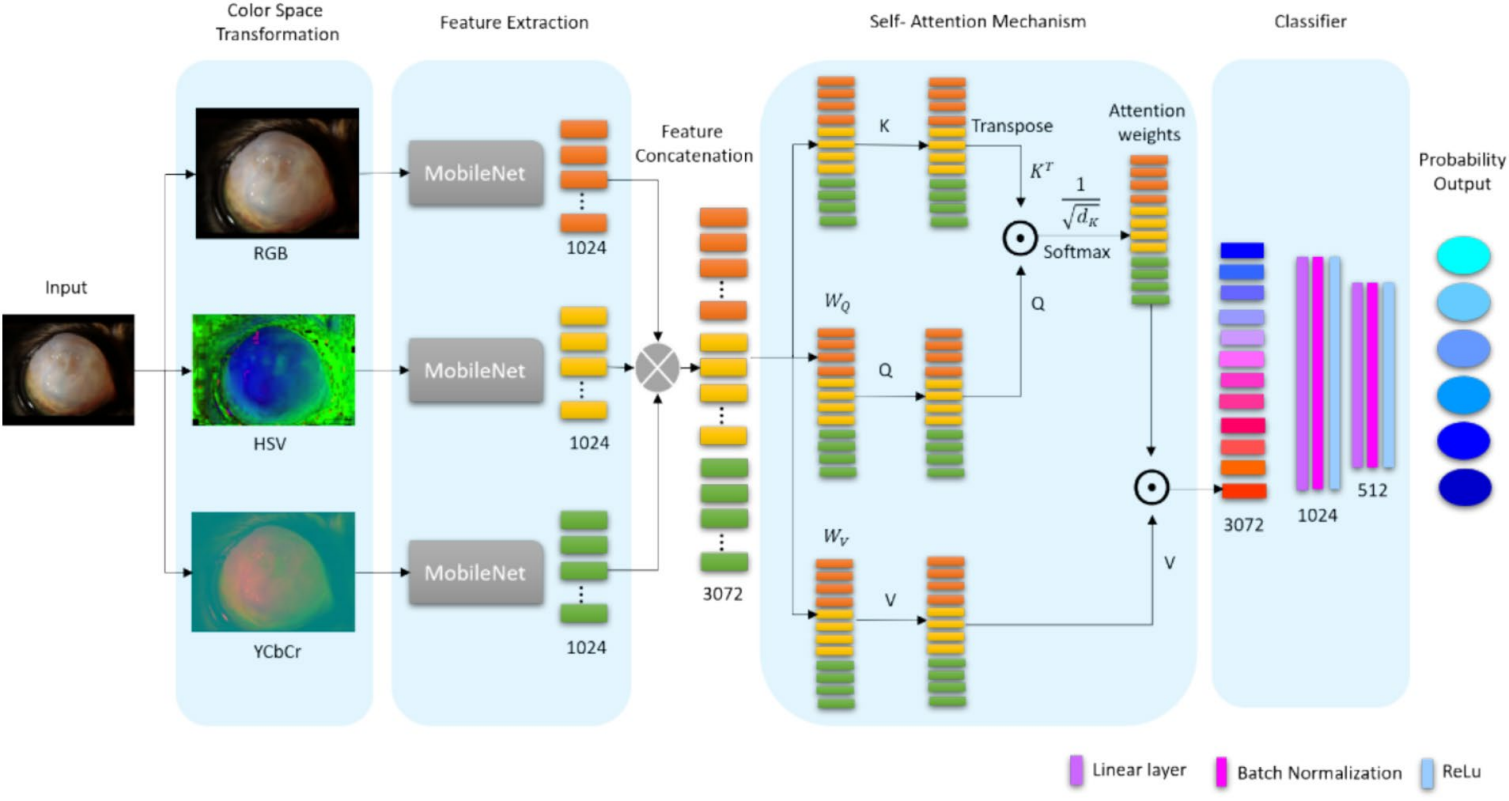
Proposed method for feline eye disease classification.

The proposed Color Space Fusion Network (CSF-Net) is designed for robust feline ocular disease classification under unconstrained, real-world imaging conditions. An overview of the proposed CSF-Net architecture is illustrated in Figure 2. The design of CSF-Net is motivated by two core challenges: (1) high visual variability caused by illumination changes and acquisition inconsistencies, and (2) strong inter-class ambiguity among visually similar ocular diseases. To address these challenges, CSF-Net integrates multi-color space feature decomposition with an attention-guided feature interaction mechanism this design enables selective emphasis on disease-relevant visual cues while suppressing illumination-induced artifacts.

Given an input feline eye image captured under real-world conditions, the image is first transformed into three complementary color spaces: RGB, HSV, and YCbCr. Each color space provides a distinct representation of chromatic and luminance information. This allows the model to disentangle disease-related visual patterns from illumination-dependent variations.

For each color space, an independent convolutional backbone is employed to extract high-level feature representations. These feature vectors are subsequently fused and refined using a self-attention mechanism, which explicitly models cross-color feature interactions. Finally, the refined feature representation is fed into a classification head to produce disease probability scores. This architecture moves beyond simple color-space concatenation by enabling attention-guided feature reasoning across heterogeneous color representations.

### 2.3 Multi-color space and feature fusion decomposition

Let $I\in {\mathbb{R}}^{H\times W\times 3}$ denote an input RBG image. The image is transformed into HSV and YCbCr color spaces using standard color conversion operations, resulting in three representations as shown in Equation 1:


$$I^{\mathrm{RGB}},I^{\mathrm{HSV}},I^{\text{YCbCr}}$$ (1)

Each color space emphasizes different visual characteristics: RGB preserves raw color intensity, HSV decouples chromatic information from luminance, and YCbCr separates luminance from color-difference components. This decomposition facilitates the extraction of complementary features that are difficult to capture within a single RGB representation. Such complementary representations are particularly relevant for feline ocular disease analysis, where subtle variations in redness, opacity, and texture are key diagnostic indicators. HSV improves robustness to illumination variability by isolating color information from intensity, while YCbCr enhances structural contrast through luminance-chrominance separation, aiding in the detection of corneal irregularities and surface changes. Together, these color spaces provide a balanced and computationally efficient representation for capturing diverse visual cues under real-world imaging conditions.

Each color-space image is independently processed by a lightweight MobileNetV3-Small backbone network f(⋅) , producing feature vectors as represented in Equation 2:


$$F_{\mathrm{RGB}}=f\;(I^{\mathrm{RGB}}),F_{\mathrm{HSV}}=f\;(I^{\mathrm{HSV}}),F_{\text{YCbCr}}=f\;(I^{\text{YCbCr}})$$ (2)

Where $F\;\in \;{\mathbb{R}}^{d}$ denotes a high-level feature embedding of dimension d =1,024. Separate encoders ensure that color-specific patterns are preserved without premature mixing across color spaces. The RGB branch is initialized with ImageNet-pretrained weights, whereas the HSV and YCbCr branches are trained from random initialization. The final classification layer of each backbone is removed to obtain a 1,024-dimensional feature representation per color space.

The extracted feature vectors are concatenated to form a unified representation as given in Equation 3:


$$F=[F_{\mathrm{RGB}};F_{\mathrm{HSV}};F_{\text{YCbCr}}]\;\in \;{\mathbb{R}}^{3d}$$ (3)

Resulting in a fused feature vector of dimension 3,072. To effectively integrate information from multiple color spaces, CSF-Net employs a single-head global self-attention mechanism that explicitly models interactions among color-specific feature representations Self-attention is based on the scaled dot-product attention mechanism (17) and is used to enhance dependencies within the fused representation. It dynamically allocates weights to the concatenated feature representation, allowing the model to focus on highly relevant features. Given query, key K , and value V , the projections are computed as presented in Equation 4:


$$Q=FW_{q},K=FW_{k},V=FW_{v}$$ (4)

Where $W_{q}$ , $W_{k}$ and $W_{v}$ are learnable projection matrices. The attention scores are computed using the scaled dot-product attention mechanism as formulated in Equation 5:


$$\text{Attention}\;(Q,K,V)=\text{softmax}\;(\frac{Q\;K^{T}}{\sqrt{d_{k}}})\;V$$ (5)

Where $d_{k}$ represents the feature dimension, ensuring numerical stability. This mechanism enables selective emphasis of disease-relevant feature interactions across color spaces while suppressing redundant or illumination-sensitive components.

Unlike channel-wise attention modules that operate within a single feature domain, the proposed self-attention mechanism facilitates cross-color reasoning. This allows CSF-Net to capture complex dependencies between heterogeneous color representations.

### 2.4 Classification head and loss function

The classification module consists of fully connected layers with batch normalization, ReLu activation and dropout regularization, followed by a final linear layer. Softmax is applied implicitly during training through the CrossEntropyLoss function. This loss function quantifies the difference between the predicted probability distribution and the true class labels. It encourages higher probabilities for the correct class while penalizing incorrect predictions. During inference, Softmax is explicitly applied to generate probability distributions over six classes: Blepharitis, Conjunctivitis, Corneal Sequestrum, Non-ulcerative Keratitis, Corneal Ulcer, and Healthy Eyes. The final classification is computed using the following formula as described in Equation 6:


$$\hat{y}=\text{softmax}(f_{\mathrm{MLP}}(F))$$ (6)

Where 𝐹 represents the refined feature vector post-attention. The weighted Cross-Entropy Loss (18) function is defined as indicated in Equation 7:


$$L=-\frac{1}{N}\;\sum_{n=1}^{N}w_{y_{n}}\;\log(\frac{\exp(z_{n,y_{n}})}{\sum_{c=1}^{C}\exp(z_{n,c})})$$ (7)

Where class weights are defined as shown in Equation 8:


$$w_{c}=\frac{\frac{1}{N_{C}}}{\sum_{j=1}^{C}\frac{I}{N_{j}}}\cdot C$$ (8)

Where 𝑁 denotes the total number of training samples, 𝐶 represents the number of classes, and $N_{C}$ indicates the number of samples belonging to class 𝑐. The term $z_{n,c}$ corresponds to the logit output for class 𝑐 of the 𝑛-th sample, while $y_{n}$ denotes the ground-truth class label of the 𝑛-th sample. The predicted class probability is computed using the Softmax function. The weight $w_{c}$ represents a normalized inverse-frequency class weight used to address class imbalance. This formulation enables more balanced learning across classes and supports robust prediction under class imbalance.

### 2.5 Experimental setup and performance metrics

The model is trained on an NVIDIA A100 Tensor Core graphics processing unit featuring Multi-Instance GPU with 7 GB memory. It was implemented using the PyTorch framework with torch vision for pretrained architectures, PIL for image handling, and scikit-learn for performance evaluation. A five-fold stratified cross-validation strategy was employed, resulting in an approximately 80:20 split between training and validation data in each fold while preserving class distributions across all disease categories. Model training was conducted for a maximum of 50 epochs with a batch size of 16, using the Adam optimizer with a learning rate of 0.0001. Early stopping was applied to prevent overfitting, with a patience of 10 epochs based on validation loss improvement. Model performance was evaluated using accuracy (Equation 9), precision (Equation 10), recall (Equation 11), F1-score (Equation 12), and macro-averaged AUC, defined, respectively, as:


$$\text{Accuracy}=\frac{\left(\mathrm{TP}+\mathrm{TN}\right)}{\left(\mathrm{TP}+\mathrm{TN}+\mathrm{FP}+\mathrm{FN}\right)}$$ (9)

$$\text{Precision}=\frac{\mathrm{TP}\;}{\mathrm{TP}+\mathrm{FP}\;}$$ (10)

$$\text{Recall}=\frac{\mathrm{TP}\;}{\mathrm{TP}+\mathrm{FN}}$$ (11)

$$F1=2\times \frac{\text{Precision}\times \text{Recall}}{\text{Precision}+\text{Recall}}$$ (12)

And AUC computed using a one-vs-one strategy for multi-class classification. The model achieving the highest validation F1-score was saved for each fold, while previous checkpoints were discarded to conserve storage. Training and validation losses and metrics were logged at each epoch and saved to disk for analysis. Reproducibility was supported through fixed random seed for cross-validation splits and by computing normalization statistics exclusively from the training data to prevent information leakage. No separate external test set was used; instead, model performance was evaluated by aggregating results across all cross-validation folds.

## 3 Results

### 3.1 Disease classification task

To evaluate the effectiveness of the proposed CSF-Net, we compared its performance against three widely used deep learning architectures, ResNet50 (19), EfficientNet (20), and ViT/16-b (21), which represent distinct points along the accuracy-computational complexity spectrum. These models were selected to assess whether the proposed framework achieves a favorable trade-off between diagnostic performance and computational efficiency, as summarized by parameter counts and FLOPs in Table 1. Table 1 focuses on backbone architectures to justify the selection of a lightweight design for CSF-Net. The full CSF-Net model with MobileNetV3-small (22) has has approximately 36.55 M parameters and 216.35 GFLOPs, reflecting the additional cost of multi-branch color-space processing and attention-based feature interaction.

**Table 1:** Comparison of deep learning models based on the number of parameters and floating-point operations (FLOPs), where lower values indicate reduced computational complexity.

Model	Parameters (M) (↓)	FLOPs (G) (↓)
Resnet50 (19)	23.52	4.13
EfficientNet (20)	4.02	0.41
ViT/16-b (21)	57.3	11.29
MobileNetV3-small (22)	**1.52**	**0.06**

Bold values indicate the best performance.

The performance comparison in Table 2 highlights the clear superiority of CSF-Net, which outperforms all baselines with the highest accuracy (0.8305), macro F1-score (0.7916), and mean fold-wise macro-AUC across five cross-validation folds (0.9690). The proposed model demonstrates a clear advantage in feline ocular disease classification, likely due to its self-attention mechanisms on multi-color space embedding, which enhance its ability to learn discriminative features across varying lighting conditions. The superior mean fold-wise macro-AUC across five cross-validation folds of 0.9690 further indicates its robustness in distinguishing between disease states, making it a promising tool for AI-driven veterinary ophthalmology applications. In addition, a t-test was conducted to determine the statistical significance of our model’s performance against that of the competing models.

**Table 2:** Performance results for classification task.

Model	Accuracy (↑)	Precision (↑)	Recall (↑)	F1 (↑)	mAUC (↑)
Resnet50	0.6113 ± 0.0037*	0.5728 ± 0.0041*	0.5855 ± 0.0083*	0.5613 ± 0.0036*	0.8950 ± 0.0020*
EfficientNet	0.6055 ± 0.0026*	0.5703 ± 0.0052*	0.5853 ± 0.0067*	0.5605 ± 0.0068*	0.8931 ± 0.0026*
ViT/16-b	0.6099 ± 0.0050*	0.5698 ± 0.0156*	0.5756 ± 0.0148*	0.5570 ± 0.0105*	0.8913 ± 0.0053*
**CSF-Net (our model)**	**0.8305 ± 0.0134**	**0.8009 ± 0.0178**	**0.8143 ± 0.0134**	**0.7916 ± 0.0077**	**0.9690 ± 0.0048**

*Marked as statistically significant with a *p*-value <0.0001. Bold values indicate the best performance.

To further investigate the impact of multi-color-space representations on classification performance, an ablation study was conducted comparing models trained using only the RGB color space with those incorporating RGB, HSV, and YCbCr inputs. As shown in Table 3, naive multi-color-space fusion resulted in only marginal performance changes across both ResNet50 and MobileNetV3-Small backbones. ResNet50 was included as a representative high-capacity convolutional baseline to assess whether color space fusion alone could improve performance independent of architectural efficiency constraints, while MobileNetV3 Small reflects a lightweight design intended for mobile deployment. Paired t-tests comparing each backbone with and without color-space fusion revealed no statistically significant differences across all evaluation metrics (*p* > 0.05), indicating that the direct inclusion of additional color spaces alone is insufficient to yield meaningful performance improvements. This indicates that complementary information across color spaces is not effectively exploited through naive fusion, thereby motivating the need for the proposed self-attention mechanism to selectively integrate and enhance cross-domain features. To better understand this behavior, the statistical properties of each color space were analyzed by computing per-channel means and standard deviations over the training set is provided in the Supplementary Figure S2.

**Table 3:** Ablation study on the effect of color space.

Backbone	RGB	Accuracy (↑)	Precision (↑)	Recall (↑)	F1 (↑)	mAUC (↑)
Resnet50	RGB only	0.6113 ± 0.0037	0.5728 ± 0.0041	0.5855 ± 0.0083	0.5613 ± 0.0036	0.8950 ± 0.0020
	RGB + HSV + YCbCr	**0.6166 ± 0.0048**	**0.5772 ± 0.0102**	**0.5918 ± 0.0068**	**0.5668 ± 0.0048**	**0.8959 ± 0.0018**
Mobilenetv3 small	RGB only	0.5942 ± 0.0066	0.5548 ± 0.0064	0.5713 ± 0.0086	0.5489 ± 0.0075	0.8846 ± 0.0031
	RGB + HSV + YCbCr	0.5936 ± 0.0055	0.5559 ± 0.0071	0.5741 ± 0.0089	0.5491 ± 0.0053	0.8888 ± 0.0041

Bold values indicate the best performance.

In the subsequent ablation study, we introduce self-attention mechanisms on multi-color space features with same backbone, same inputs to the same training protocol and it indicates a more pronounced improvement in classification performance. Table 4 demonstrates a significant boost in performance with the addition of self-attention. Compared to the baseline, incorporating self-attention raises accuracy to 0.8304 and mean macro-AUC (5-fold CV) to 0.9690, with consistent improvements across precision, recall, and F1-score. This confirms the effectiveness of attention in leveraging multi-color space features and enhancing classification accuracy.

**Table 4:** Ablation study on the effect of incorporating self-attention.

Mode	Accuracy (↑)	Precision (↑)	Recall (↑)	F1 (↑)	mAUC (↑)
w/o attention	0.5936 ± 0.0055*	0.5559 ± 0.0071*	0.5741 ± 0.0089*	0.5491 ± 0.0053*	0.8888 ± 0.0041*
W attention	**0.8305 ± 0.0134**	**0.8009 ± 0.0178**	**0.8143 ± 0.0134**	**0.7916 ± 0.0077**	**0.9690 ± 0.0048**

*Marked as statistically significant with a *p*-value <0.0001. Bold values indicate the best performance.

**Figure 3:**
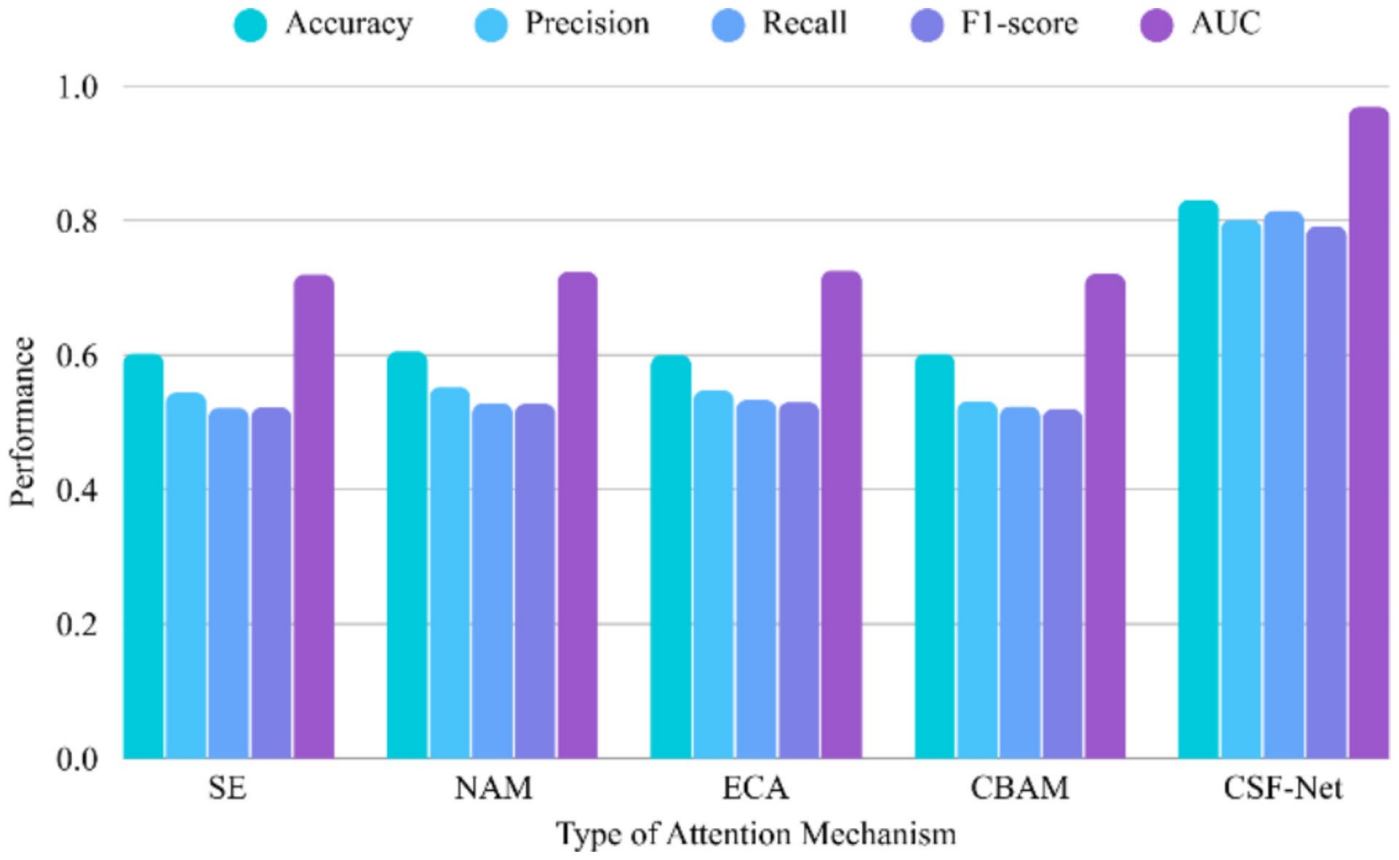
Ablation study evaluating the impact of different types of attention mechanisms.

Figure 3 presents the results of an ablation study evaluating the impact of incorporating different types of attention mechanisms into the model. Among the methods tested, Self-Attention achieved the highest performance across all evaluation metrics, with notable improvements. In contrast, conventional lightweight attention modules such as SE (23), CBAM (Channel Only) (24), NAM (25), and ECA (26) demonstrated relatively similar performance, with accuracy scores hovering around 0.60 and mean AUC values between 0.7196 and 0.7256. Although ECA and NAM slightly outperformed SE in recall and F1-score, the overall improvements were marginal. These results highlight the superior capability of Self-Attention in capturing complex dependencies, thereby enhancing classification performance more effectively than simpler channel-wise attention mechanisms.

**Figure 4:**
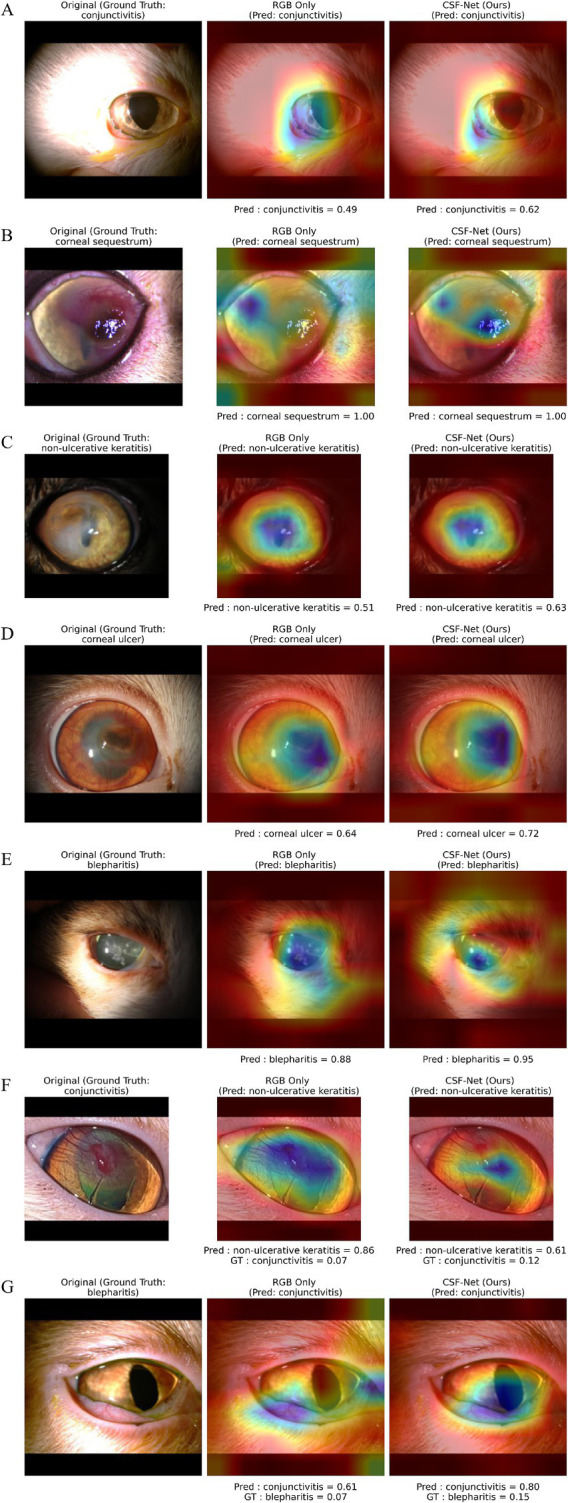
Qualitative comparison between RGB-only and CSF-Net predictions using Grad-CAM visualizations. Each row **(A–G)** shows the original image (left), RGB-only model output (middle), and CSF-Net output (right), along with predicted class probabilities. **(A–E)** Represent correctly classified cases, where CSF-Net demonstrates higher prediction confidence and more focused attention on clinically relevant regions compared to the RGB baseline. **(F,G)** Illustrate misclassified cases involving visually overlapping conditions, where both models fail; however, CSF-Net produces more informative probability distributions by assigning higher probabilities to the correct class and reducing overconfident incorrect predictions.

To further interpret the decision-making process of our model, we employ Gradient-weighted Class Activation Mapping (Grad-CAM) (27) to visualize salient regions in feline ocular disease classification. The activation maps are superimposed on the original eye images, highlighting the regions that contribute most to the model’s predictions in Figure 4. These visualizations help validate the clinical relevance of the features learned by the model and ensure that it focuses on meaningful anatomical structures for disease identification.

Figures 4F,G illustrates representative misclassified cases visualized using Grad-CAM, which are consistent with the quantitative misclassification patterns observed across disease categories. Conditions such as blepharitis, conjunctivitis, non-ulcerative keratitis, and corneal ulcer exhibited lower classification accuracy due to substantial clinical overlap in visual manifestations, including periocular inflammation, conjunctival hyperemia, corneal haze, and diffuse redness. Blepharitis was frequently confused with conjunctivitis and non-ulcerative keratitis, reflecting shared inflammatory features around the eyelids and ocular surface, while conjunctivitis was often misclassified as blepharitis or keratitis due to overlapping conjunctival and corneal involvement. Corneal ulcer cases were occasionally misidentified when ulcer margins were subtle or visually dominated by surrounding inflammation. In contrast, regular eyes showed high classification accuracy, indicating reliable discrimination in the absence of pathology. Overall, the Grad-CAM visualizations demonstrate that misclassifications primarily arise from clinically plausible feature overlap rather than erroneous model focus, highlighting the inherent diagnostic challenges in visually similar feline ocular diseases. These results highlight the effectiveness of the proposed model in recognizing distinct pathological features in most classes, while also indicating the need for further refinement to improve classification performance in more ambiguous cases.

**Figure 5:**
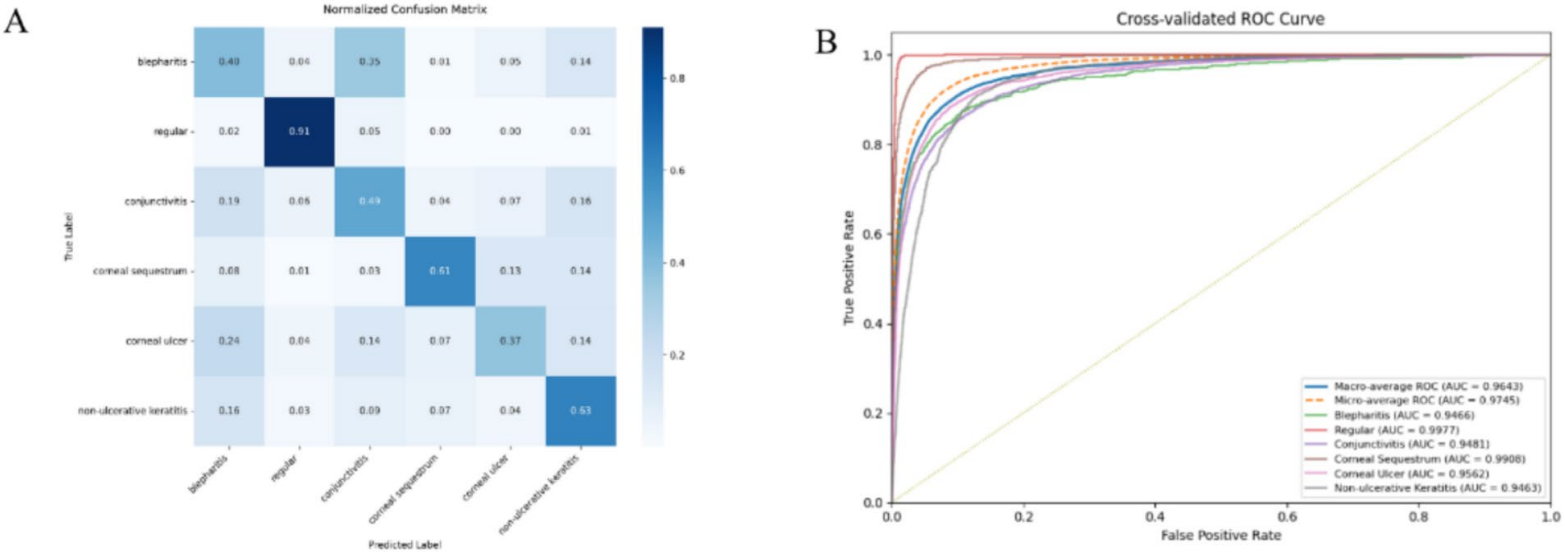
**(A)** Normalized confusion matrix of CSF-Net showing class-wise performance and common misclassifications among visually similar disease categories. **(B)** Cross-validated ROC curves with macro-, micro-, and per-class AUCs, indicating strong class discrimination (macro-AUC = 0.964).

To further evaluate the model’s classification performance across different disease categories, a normalized confusion matrix was generated (Figure 5A). Table 5 represents the class-wise summary of the classification result. The model achieved the highest classification accuracy for regular eyes (91.0%), indicating robust discrimination between healthy and diseased cases. In contrast, inflammatory and corneal conditions such as blepharitis (40.0%), conjunctivitis (49.0%), and corneal ulcer (37.0%) showed lower accuracy and were frequently misclassified among each other, reflecting substantial visual and clinical overlap. Corneal sequestrum (61.0%) and non-ulcerative keratitis (63.0%) demonstrated moderate performance, with misclassifications primarily occurring between corneal disease categories with similar opacity and inflammatory features.

**Table 5:** Class-wise normalized confusion matrix summary.

True label	Correct classification (%)	Most frequent misclassifications (%)
Blepharitis	40.00%	Conjunctivitis (35.0%), Non-Ulcerative Keratitis (14.0%)
Regular	91.00%	Conjunctivitis (5.0%), Blepharitis (2.0%)
Conjunctivitis	49.00%	Blepharitis (19.0%), Non-Ulcerative Keratitis (16.0%)
Corneal sequestrum	61.00%	Non-Ulcerative Keratitis (14.0%), Corneal Ulcer (13.0%)
Corneal ulcer	37.00%	Blepharitis (24.0%), Conjunctivitis (14.0%)
Non-Ulcerative Keratitis	63.00%	Blepharitis (16.0%), Conjunctivitis (9.0%)

### 3.2 Mobile application

To enhance accessibility to feline ocular disease detection, we developed PurrfectEyes, a mobile application that assists pet owners in monitoring their cat’s eye health using smartphone images. The application integrates an AI-assisted preliminary screening system capable of identifying five common feline ocular diseases including blepharitis, conjunctivitis, corneal sequestrum, non-ulcerative keratitis, and corneal ulcer, along with healthy cases. Built on a deep learning model that incorporates multiple color space representations and a self-attention mechanism, PurrfectEyes processes eye images to generate disease probability scores and visual explanations using Grad CAM. In addition, an automated medical report generation module structures diagnostic findings, patient information, and recommendations, with text enhanced using GPT 3.5 for improved readability, and exports the results as securely stored PDF reports. The system architecture of the proposed mobile application and example diagnostic report outputs with Grad-CAM visualizations are provided in the Supplementary Figures S3, S4, respectively.

**Figure 6:**
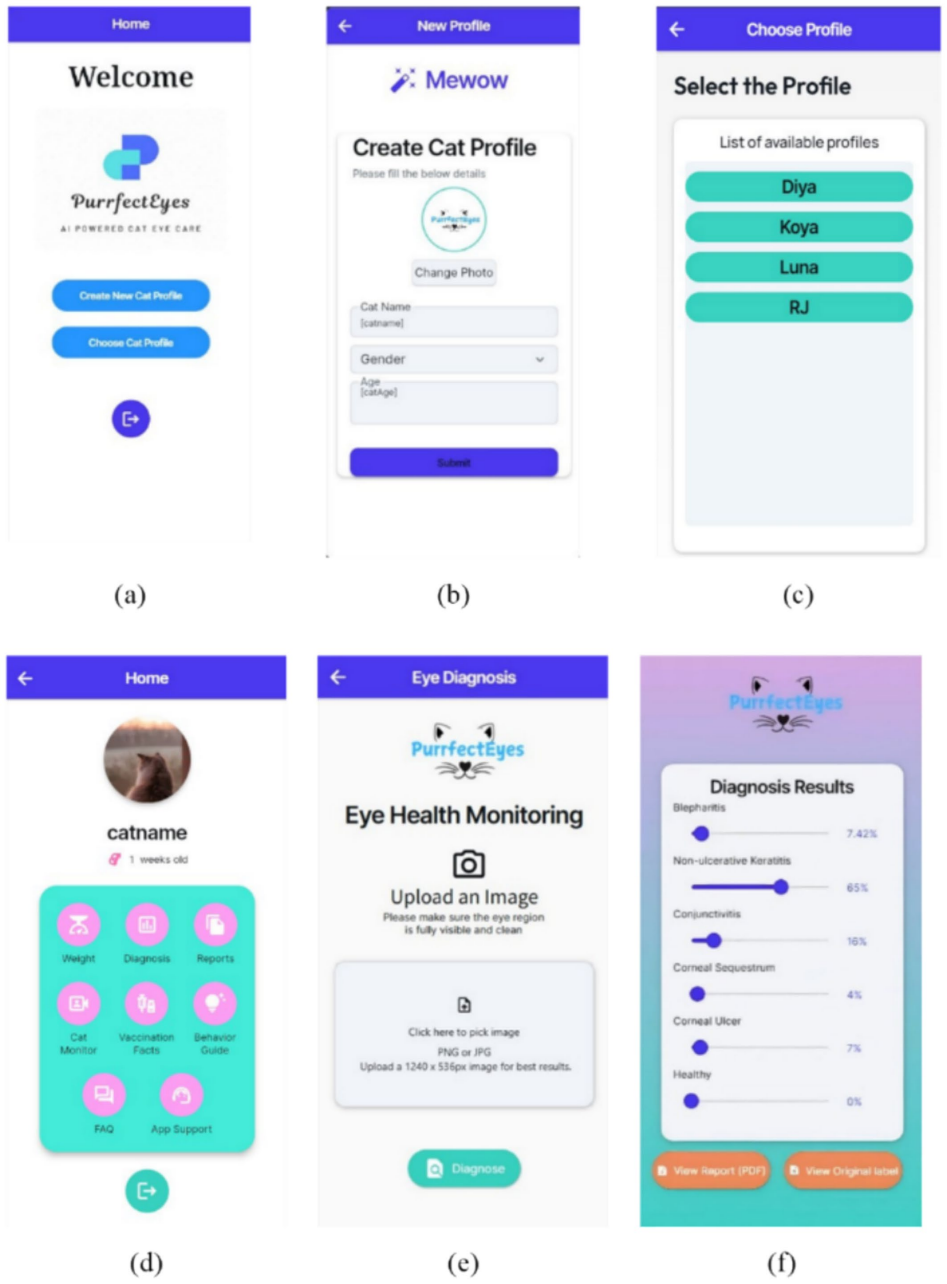
Interface of mobile application—PurrfectEyes: **(a)** home screen, **(b)** create new cat profile, **(c)** choose from existing cat profiles, **(d)** cat profile home, **(e)** eye diagnosis screen, **(f)** diagnosis result screen.

PurrfectEyes mobile application provides a seamless and user-friendly interface for feline eye disease monitoring and diagnosis. Figure 6 illustrates key screens of the application. (a) Displays the home screen, allowing users to create or select a cat profile. (b) Shows the new cat profile creation screen, where users input details such as name, age, and gender. (c) Presents the profile selection interface, listing previously registered cats for easy access. (d) Represents the cat profile home screen, offering navigation to essential features such as weight tracking, disease diagnosis, medical reports, vaccination facts, behavior guides, cat monitoring, and app support. These additional functionalities enhance the application’s usability by providing a comprehensive health management system for pet owners. (e) is the eye diagnosis screen, where users can upload an image of their cat’s eye for AI-based analysis. Finally, (f) showcases the diagnosis results screen, providing probability-based disease predictions with an option to generate a detailed PDF report. This intuitive design ensures accessibility for pet owners while integrating advanced AI-based diagnostics.

## 4 Discussion

Feline ocular diseases present a significant challenge in veterinary medicine due to their impact on vision and overall feline health, as well as the difficulty of early diagnosis in non-specialized settings. The application of artificial intelligence in veterinary ophthalmology has expanded significantly with advances in deep learning-based image analysis. Early studies primarily focused on controlled clinical imaging tasks, such as radiographic and ophthalmic image analysis using standardized acquisition protocols (4). In human ophthalmology, deep learning models have demonstrated strong performance in detecting retinal diseases, including diabetic retinopathy and age-related macular degeneration (13–15). However, these approaches generally assume high-quality, standardized imaging conditions. This limits their applicability to real-world scenarios characterized by variability in lighting, viewpoint, and acquisition devices.

In veterinary contexts, AI-based approaches have been increasingly explored, but most existing work focuses on canine ocular diseases (2, 5), with limited studies addressing feline-specific conditions. Due to anatomical and physiological differences between canine and feline eyes, including variations in corneal structure and low-light adaptation, models trained on canine data do not readily generalize to feline applications (5). Furthermore, existing feline-focused studies often rely on small-scale datasets or limited disease categories, which restricts robustness and generalizability (28). Recent approaches using object detection frameworks, such as YOLO, demonstrate feasibility for real-time disease detection (29). However, they remain limited in capturing fine-grained pathological differences required for distinguishing visually similar ocular conditions.

Beyond veterinary applications, prior work has explored the use of alternative color spaces, such as HSV and YCbCr, to improve robustness to illumination variability (30). While multi-color representations can enhance feature diversity, most existing approaches rely on simple concatenation or parallel processing. These methods do not explicitly model interactions between heterogeneous feature domains. In parallel, attention mechanisms have emerged as effective tools for improving feature representation in medical imaging tasks (19–21, 22). Lightweight modules such as SE (23), CBAM (24), NAM (25), and ECA-Net (26) primarily focus on channel-wise or spatial feature recalibration within a single representation space. Although effective, they are not designed to capture interactions across multiple feature domains. In contrast, self-attention mechanisms have demonstrated strong capability in modeling complex relationships and long-range dependencies (17), yet their integration with multi-color feature representations remains underexplored in veterinary ophthalmology.

These limitations highlight a gap in existing work. Current approaches either rely on single-color representations, assume controlled imaging conditions, or lack mechanisms to explicitly model interactions across heterogeneous feature spaces. The proposed CSF-Net addresses this gap by introducing an attention-guided multi-color feature interaction framework. This enables more effective integration of complementary color-space information and improves discrimination under real-world imaging conditions. Unlike prior methods, the proposed approach moves beyond independent feature extraction or naive fusion by enabling dynamic interaction across heterogeneous color-space representations.

The ablation results demonstrate that multi-color representations alone result in only marginal or inconsistent performance changes, indicating that simple fusion is insufficient. This observation is further supported by the distribution analysis presented in the Supplementary material, which shows substantial overlap across color spaces. This suggests that complementary information is not explicitly separable through direct concatenation. As a result, additional color-space information remains underutilized without a mechanism to selectively emphasize informative features. However, when combined with attention-based interaction, performance improves significantly. This highlights the importance of modeling relationships across color domains to effectively exploit complementary visual cues.

The experimental results show that CSF-Net outperforms established architectures such as ResNet50, EfficientNet, and ViT, achieving superior accuracy and a macro-AUC of 0.964. The ROC analysis in Figure 5B further confirms strong class discrimination across all disease categories. This indicates that the model effectively separates classes across decision thresholds rather than relying on a fixed boundary. The improvement can be attributed to the proposed attention-guided fusion, which enables selective integration of complementary information while suppressing redundant or less informative signals.

In addition, Figure 4 provides qualitative comparisons with an RGB-only baseline. CSF-Net produces higher prediction confidence and more focused attention on clinically relevant regions, whereas the RGB model exhibits more diffuse attention patterns. This suggests improved localization of disease-specific features through the use of diverse color representations. Analysis of misclassified cases (Figures 4F–G) further shows that, even when incorrect predictions occur, CSF-Net assigns higher probabilities to the correct class and reduces overconfident errors. This indicates more informative ranking of disease classes, which contributes to the high AUC. Such behavior is particularly important for visually overlapping conditions such as blepharitis, conjunctivitis, and keratitis, where subtle differences must be captured for reliable discrimination.

Despite these contributions, several limitations should be acknowledged. First, class ambiguity remains a significant challenge, as multiple ocular diseases share overlapping visual characteristics. This makes precise discrimination difficult even for expert clinicians. Second, the dataset composition introduces potential sources of bias, including imbalance across disease categories and variability in imaging devices and acquisition conditions. Third, the dataset lacks subject-level identifiers, and cross-validation was performed at the image level, which may introduce a risk of data leakage. Additionally, the dataset is derived primarily from a single geographic region, which may limit generalizability to diverse populations and clinical environments. The use of single-label annotations further simplifies inherently complex clinical scenarios where multiple conditions may co-occur. No explicit correlation analysis between breed, imaging environment, and disease labels was conducted. These factors should be considered when interpreting performance results.

Future work will address these limitations by expanding the dataset to include a broader range of diseases, imaging conditions, and multi-center data sources. Incorporating subject-level identifiers will enable more robust evaluation protocols and reduce potential leakage effects. Multi-label learning approaches will be explored to better reflect real-world clinical complexity. In addition, integrating contextual information, such as breed and demographic data, may further improve diagnostic performance. External validation on independent datasets will be essential to confirm the generalizability and clinical applicability of the proposed method.

## 5 Conclusion

Artificial intelligence has strong potential to assist in early screening and recognition of veterinary diseases using everyday imaging devices. In this study, we proposed CSF-Net, an attention-guided multi-color space fusion framework designed for automated classification of feline ocular diseases under real-world imaging conditions. By combining complementary color space representations (RGB, HSV, and YCbCr) with a self-attention mechanism, the model improves feature learning and reduces the impact of illumination variability commonly present in pet-owner captured images. Experimental results demonstrate that CSF-Net consistently outperforms representative deep learning architectures, including ResNet50, EfficientNet, and ViT-B16, while ablation studies confirm that performance improvements primarily arise from attention-guided feature interaction rather than color space expansion alone. The model was further integrated into the PurrfectEyes mobile application to support preliminary screening of feline eye diseases using smartphone images. The system provides probability-based predictions and visual explanations to assist pet owners in recognizing potential abnormalities and seeking timely veterinary consultation. However, additional data and broader disease coverage are required to further improve model generalization and performance in visually ambiguous conditions. Future work will focus on expanding datasets, refining disease differentiation, and improving real-world deployment to support more reliable AI-assisted feline ocular health monitoring.

## Data Availability

The original contributions presented in the study are included in the article/Supplementary material, further inquiries can be directed to the corresponding author.
