# Supplementary material for: CSF-net: a color space fusion network with self-attention-driven feature learning for feline ocular diseases classification
Source: Front Vet Sci. 2026 Apr 22;13:1826139. doi: 10.3389/fvets.2026.1826139 (PMC13143524; doi:10.3389/fvets.2026.1826139)
Supplement: Supplementary file 1 [file Data_Sheet_1.PDF]

Supplementary Material

S1. Dataset Description

Figure S1 (A) illustrates the age distribution of the sampled feline population, highlighting a significant concentration of young cats, particularly those aged 1 year (4734 samples), followed by newborn kittens (0 years, 1793 samples), indicating a higher availability or diagnostic need for younger cats. Figure S1 (B) presents the breed distribution, revealing that Korean Shorthair (35.5%) and Persian (22%) cats constitute the majority of the dataset. The dataset includes a total of 24 breeds, with Exotic Shorthair (5.6%), Scottish Fold (4.9%), and Abyssinian (4.8%) also being significantly represented, indicating a diverse sample of feline populations.

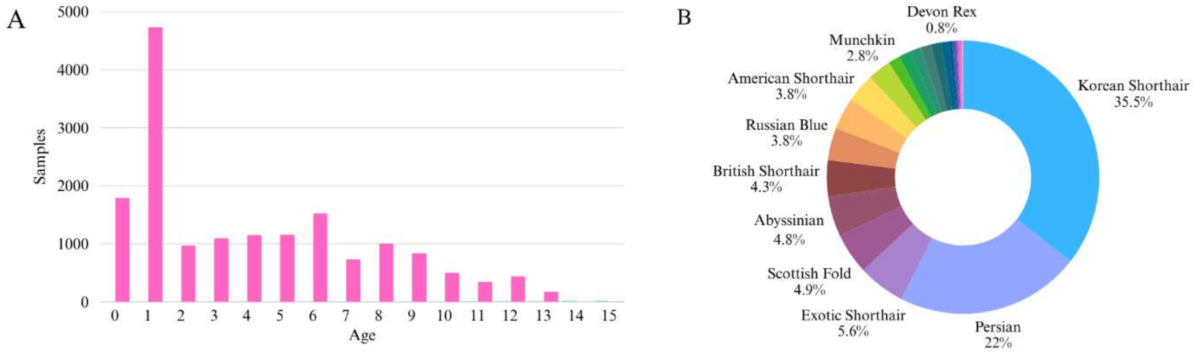

Figure S1. Dataset distribution. (A) Age distribution showing a predominance of younger cats, especially at 1 year and 0 years. (B) Breed distribution with Korean Shorthair and Persian as the most represented breeds, followed by several minority breeds.

S2. Color Space Analysis

To investigate the behaviour of different color representations, the statistical properties of RGB, HSV, and YCbCr color spaces were analysed by computing per-channel means and standard deviations over the training dataset. Figure S2 illustrates the normalized value distributions for RGB, HSV, and YCbCr channels, modelled using Gaussian approximations defined by their empirical mean (μ) and standard deviation (σ). The RGB channels exhibit broad distributions with substantial overlap, reflecting high variance and redundancy across color channels. Although the HSV color space partially decouples chromatic and intensity information, significant overlap persists among the H, S, and V channels, limiting their discriminative separation when used in isolation. In contrast the YCbCr color space, particularly the chrominance channels (Cb and Cr) show more compact distributions with reduced variance, suggesting increased stability and potential discriminative capacity.

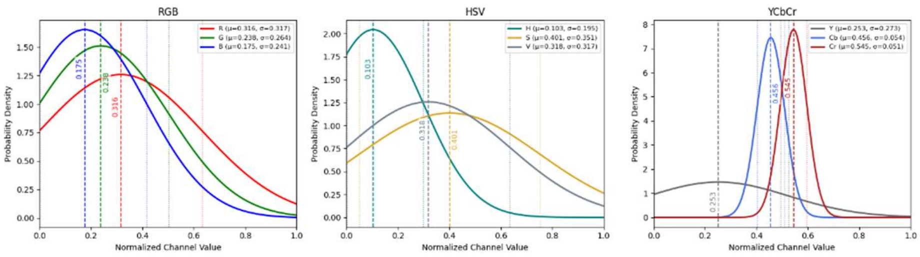

Figure S2. Per-channel normalized value distributions for RGB, HSV, and YCbCr color spaces computed over the training set. Each curve represents a Gaussian approximation defined by the empirical mean (μ) and standard deviation (σ) of the corresponding channel. Dashed vertical lines indicate channel means, while dotted lines denote ±1σ. The distributions reveal substantial overlap among RGB and HSV channels, whereas YCbCr exhibits more compact chrominance distributions (Cb, Cr) with reduced variance, suggesting increased stability but limited discriminative separation when used in isolation.

Despite these differences, Figure S2 also reveals persistent overlap across all color spaces, indicating that complementary information is not explicitly separable through simple concatenation. As a result, the additional color-space information remains largely underutilized in the absence of a mechanism capable of selectively emphasizing informative features while suppressing redundant signals. This observation explains the limited performance gains observed with naive multi-color-space fusion in Table 3. Importantly, these findings clarify that multi-color-space representations should be interpreted as a source of latent discriminative potential rather than a direct performance enhancement. The substantial and consistent improvements achieved by CSF-Net demonstrate that the primary contribution arises from the attention mechanism, which enables dynamic weighting and interaction across color spaces.

S3. Mobile Application:

S3.1. System Architecture

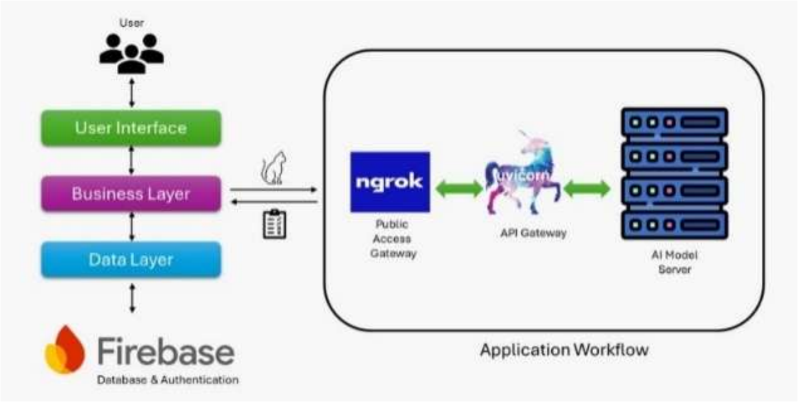

Figure S3. System Architecture of "PurrfectEyes" Mobile Application

Figure S3 presents the overall system architecture of PurrfectEyes, illustrating the interaction between the mobile interface and the backend inference pipeline. User captured eye images are transmitted to a remote server via secure API endpoints enabled by Ngrok and Uvicorn, where model inference is performed with an average processing time of 57.31 milli second per image. This design delivers timely diagnostic results while minimizing computational requirements on the client side. The backend inference engine is implemented in PyTorch, and the mobile interface is developed using Flutter with Firebase supporting data storage and management.

S3.2. Diagnostic Report Output

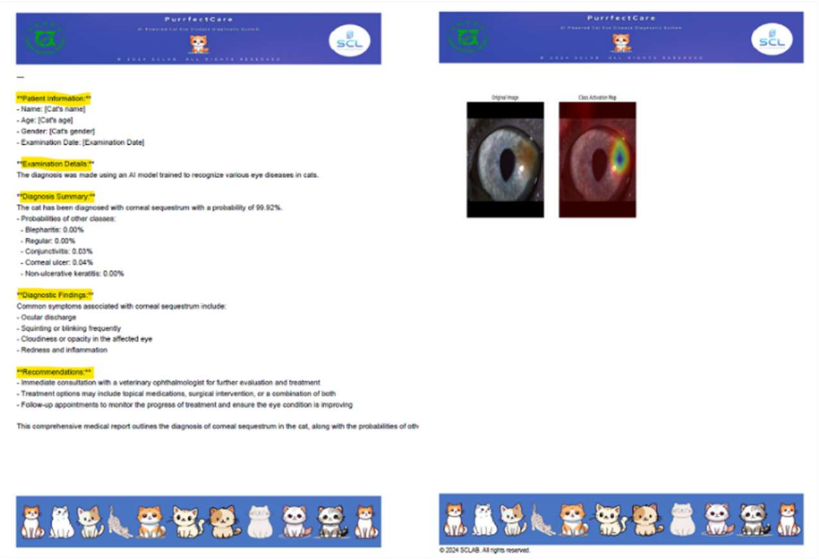

PurrfectCare

Patient Information:
- Name: [Cat's name]
- Age: [Cat's age]
- Gender: [Cat's gender]
- Examination Date: [Examination Date]

Examination Details:
The diagnosis was made using an AI model trained to recognize various eye diseases in cats.

Diagnosis Summary:
The cat has been diagnosed with corneal sequestrum with a probability of 99.92%.
- Probabilities of other classes:
  - Blepharitis: 0.00%
  - Regular: 0.00%
  - Conjunctivitis: 0.03%
  - Corneal ulcer: 0.04%
  - Non-ulcerative keratitis: 0.00%

Diagnostic Findings:
Common symptoms associated with corneal sequestrum include:
- Ocular discharge
- Squinting or blinking frequently
- Cloudiness or opacity in the affected eye
- Redness and inflammation

Recommendations:
- Immediate consultation with a veterinary ophthalmologist for further evaluation and treatment
- Treatment options may include topical medications, surgical intervention, or a combination of both
- Follow-up appointments to monitor the progress of treatment and ensure the eye condition is improving

This comprehensive medical report outlines the diagnosis of corneal sequestrum in the cat, along with the probabilities of oth

PurrfectCare

Original Image

Class Activation Map

© 2024 SCLAB. All rights reserved.

Figure S4. Medical Report Generated by GPT-3.5 with which includes patient information, diagnostic findings, and recommendations along with Grad-CAM heatmaps
